# Quality measures for palliative care in patients living with cancer in low- and middle-income countries: a scoping review protocol

**DOI:** 10.1186/s13643-023-02237-x

**Published:** 2023-05-04

**Authors:** M. K. T. N. Motlana, S. E. Makhunga, N. Jafta, T. G. Ginindza

**Affiliations:** 1 Cancer & Infectious Diseases Epidemiology Research Unit (CIDERU), College of Health Sciences, University of KwaZulu-Natal, Durban, South Africa; 2 Discipline of Public Health Medicine, School of Nursing and Public Health, College of Health Sciences, University of KwaZulu-Natal, 2nd Floor George Campbell Building, Durban, 4001 South Africa; 3 Discipline of Occupational and Environmental Health, School of Nursing and Public Health, College of Health Sciences, University of KwaZulu–Natal, Durban, South Africa

**Keywords:** Palliative care, Quality measures, LMICs, Quality indicators, Patient satisfaction

## Abstract

**Introduction:**

Quality assessment is a critical component of determining the value of medical services, including palliative care. The utilisation of palliative care quality measures could assist in assessing the degree to which patients living with cancer conform to best practice of palliative care, identifying gaps and monitoring changes in cancer care delivery models in different setting. This scoping review aims to map the available data on the usage of palliative care quality indicators that are relevant to cancer patients in low- and middle-income countries (LMICs).

**Methods:**

To structure this study, we will use the framework developed by Arksey and O’Malley, the Levac et al. recommendations and the Joanna Briggs Institute recommendations. We will search EBSCOHost, Web of Science, ProQuest One Academic, MEDLINE and Google Scholar for evidence on palliative care quality measures applicable for patients living with cancer published from inception till 2022. We will search grey literature in the form of dissertations, conference proceedings and websites of international organisations such as the World Health Organisation (WHO) reporting palliative care quality measures applicable to patients living with cancer in LMICs.

**Discussion:**

The purpose of this study is to establish the extent of existing research on the palliative care quality measures in LMICs. Although palliative care is still a new phenomenon, understanding of the palliative care quality measures applicable for cancer patients will assist to improve care across all components of health systems.

**Ethics and dissemination:**

No ethical approval is required for the study as the data collection and results of the proposed scoping review will be conducted and disseminated electronically using peer-reviewed journals, print and presentations at scientific conferences and stakeholder presentations.

## Introduction

Cancer remains a worldwide public health challenge, despite the growing investments on research and development towards preventative and treatment interventions [1]. The global cancer incidence is projected to reach 30.2 million new cases and 16.3 million cancer-related deaths in year 2040 [2, 3]. The need for palliative care over the years has increased [4–6], and it has been estimated that globally 40 million people require palliative care [2]. However, about 14% of this number receive palliative care services [4, 7].

Palliative care is defined as an approach integrated to improve the quality of patients’ lives, including the role of caregivers, who are faced with various challenges (physical, social, cultural, psychological and spiritual) associated with life threatening illness [5, 8]. Over the years, international health groups such as the World Health Organization (WHO), non-governmental organisations and governments have increasingly prioritised access to cancer care especially in low- and middle-income countries (LMICs) [9, 10]. With the phenomenon being integrated into health systems, research activities in palliative medicine has been increasing exponentially over the years. According to a systematic review conducted in 2008, the number of palliative care and hospice research publications from all Ovid Medline publications rose from 0.08% in 1970 to 0.38% in 2005 [11]. A recent publication looking at the global palliative care research using the bibliometric review and mapping analysis quantified that palliative care publications increased by around fourfold from 2002 to 2020, with a 19% 5-year increase projected in 2025 [12]. However, on measuring the scope of need and effectiveness of palliative care, it has proven to be difficult to generalise outcomes when using existing methodologies [4, 13–16].

According to Kamal (2013), standardisation of palliative processes and methods to deliver state-of-the art palliative care services is critical if similar outcomes are to be achieved [17, 18]. Palliative care quality measures provide methods for measuring the frequency of achieving ideal practice, benchmarking across practices and identifying gaps in health care [19]. In palliative care, quality measures can be conducted focusing on the following: (i) structural (focusing on people, resources and assets related to care), (ii) process (how care is delivered) and (iii) outcome measures (commonly identified as those that can change a patient’s health state) [19].

The utilisation of quality measures could assist in assessing the degree to which patients living with cancer conform to best-practice palliative care and monitoring changes in cancer care delivery models in different setting [17]. The purpose of this scoping review is to map the evidence on the use of palliative care quality measures applicable to patients living with cancer through synthesis of data from qualitative and quantitative studies. The study will primarily focus on low- and middle-income countries. According to The World Bank, low- and middle-income countries are defined as developing countries with a gross national (GNI) per capita of $1036 to $12,535 [20]. It is anticipated that the findings of the study will enable the researchers to identifying gaps for quality assessment in the provision of palliative care and monitoring changes in cancer care delivery models in different setting. The results of the study will also guide policymakers in tailor making palliative care quality measures adjustable to LMICs settings.

## Methods and analysis

This study is part of a larger study exploring the barriers and facilitators to implementing a multidisciplinary approach for improved palliative care services in health facilities, KwaZulu-Natal. We will conduct a scoping review which is guided by Arksey and O’Malley [21] framework and supported by Levac et al. [22] recommendation. The Arksey and O’Malley framework comprises the following: (i) identifying the research question, (ii) identifying relevant studies, (iii) study selection, (iv) charting the data and (v) collating, summarising and reporting the results and (vi) consultation. An optional sixth stage of consultation was proposed by Arksey and O’Malley as a measure to seek insight from stakeholders beyond what was found in the literature. This scoping review will not include consultation with stakeholders as we are interested in the palliative care quality measures used and not the results/functionality of those quality measures. We followed the preferred reporting items for systematic and meta-analysis extension for protocol guidelines to develop this protocol. However, we will use the Preferred Reporting Items for Systematic Reviews and Meta-analyses (PRISMA): Extension for scoping reviews checklist to report this study results.

### Eligibility of the research question for the scoping review

The main research question is ‘What are the measures used to quantify the provision of palliative care quality for patients living with cancer and the extent of existing research available in low- and middle-income countries?’.

The research sub-questions are:

1. What are the frequently used palliative care measures used in low- and middle-income countries?
2. How much published work on palliative care quality measures in low- and middle-income countries exists?

We used the following elements: (population, concept, and context) to conceptualise the review question as depicted in Table 1.

**Table 1:** PCC framework for determining the eligibility of the studies for the primary research questions

Criteria	Determinants
Population	Both children and adult patients living with cancer (age: 0–80 + years)
Concept	Palliative care quality measures
Context	Lower and middle-income countries (LMICs)

### Identification of relevant studies

We will conduct a comprehensive search of relevant literature published from inception to July 2022. Primary studies published in peer-reviewed journals using different study designs will be included in the review study. Databases that will be used to source literature will include PubMed, EBSCOHost (Academic search complete, CINAHL with Full-text, and Health Source), Web of Science, ProQuest One Academic, Medline Ovid, Scopus, Cochrane and Google Scholar. We will also look for grey literature in government publications, university dissertations and thesis from institutional repositories and reports from international organisations such as the World Health Organization and palliative care associations (e.g. African Palliative Care Association). We will search for more studies that are relevant by manually scanning all references listed in the included papers to find studies that have not been indexed by electronic databases. The principal investigators (PIs) of the included articles will be contacted for missing data.

The comprehensive search strategy will be co-developed by the principal investigator and the university librarian to ensure the correct usage of terminology indexing and Medical Subject Headings (MeSH) terms. The search strategy will be piloted to check the appropriateness of keywords and databases; furthermore, database search combination will be recorded in Table. The following keywords or MeSH terms will be used: ‘cancer patients’, ‘palliative care quality measures’/ ‘palliative care quality metrics’ (Table 2).

**Table 2:** Proposed search terms developed on PubMed

Palliative care^a^	**palliative care:** “palliative care”[MeSH Terms] OR (“palliative”[All Fields] AND “care”[All Fields]) OR “palliative care”[All Fields] **hospice care:** “hospice care”[MeSH Terms] OR (“hospice”[All Fields] AND “care”[All Fields]) OR “hospice care”[All Fields] **end of life care:** “terminal care”[MeSH Terms] OR (“terminal”[All Fields] AND “care”[All Fields]) OR “terminal care”[All Fields] OR (“end”[All Fields] AND “life”[All Fields] AND “care”[All Fields]) OR “end of life care”[All Fields] OR “hospice care”[MeSH Terms] OR (“hospice”[All Fields] AND “care”[All Fields]) OR “hospice care”[All Fields] OR (“end”[All Fields] AND “life”[All Fields] AND “care”[All Fields])
Patients living with cancer^a^	**cancer:** “cancer’s”[All Fields] OR “cancerated”[All Fields] OR “canceration”[All Fields] OR “cancerization”[All Fields] OR “cancerized”[All Fields] OR “cancerous”[All Fields] OR “neoplasms”[MeSH Terms] OR “neoplasms”[All Fields] OR “cancer”[All Fields] OR “cancers”[All Fields] **patients:** “patient’s”[All Fields] OR “patients”[MeSH Terms] OR “patients”[All Fields] OR “patient”[All Fields] OR “patients’s”[All Fields]
Palliative care quality measures^a^	**quality:** “qualities”[All Fields] OR “quality”[All Fields] OR “quality’s”[All Fields] **measures:** “measurability”[All Fields] OR “measurable”[All Fields] OR “measurably”[All Fields] OR “measure’s”[All Fields] OR “measureable”[All Fields] OR “measured”[All Fields] OR “measurement”[All Fields] OR “measurement’s”[All Fields] OR “measurements”[All Fields] OR “measurer”[All Fields] OR “measurers”[All Fields] OR “measuring”[All Fields] OR “measurings”[All Fields] OR “measurment”[All Fields] OR “measurments”[All Fields] OR “weights and measures”[MeSH Terms] OR (“weights”[All Fields] AND “measures”[All Fields]) OR “weights and measures”[All Fields] OR “measure”[All Fields] OR “measures”[All Fields] **metrics:** “benchmarking”[MeSH Terms] OR “benchmarking”[All Fields] OR “metrics”[All Fields] OR “metric’s”[All Fields] OR “metronidazole”[MeSH Terms] OR “metronidazole”[All Fields] OR “metric”[All Fields] **patient satisfaction:** “patient satisfaction”[MeSH Terms] OR (“patient”[All Fields] AND “satisfaction”[All Fields]) OR “patient satisfaction”[All Fields] **indicators:** “indicate”[All Fields] OR “indicated”[All Fields] OR “indicates”[All Fields] OR “indicating”[All Fields] OR “indicative”[All Fields] OR “indicatives”[All Fields] OR “indicators and reagents”[Pharmacological Action] OR “indicators and reagents”[MeSH Terms] OR (“indicators”[All Fields] AND “reagents”[All Fields]) OR “indicators and reagents”[All Fields] OR “indicator”[All Fields] OR “indicators”[All Fields] OR “indice”[All Fields] OR “indices”[All Fields]
LMICs^a^	“developing countries”[MeSH Terms] OR (“developing”[All Fields] AND “countries”[All Fields]) OR “developing countries”[All Fields] OR “lmics”[All Fields] OR “lmic’s”[All Fields]

^a^Results of the three tables will be combined with “AND”

### Selection of eligible studies

Relevant studies will be selected using the following criteria:

### Inclusion criteria

The inclusion criteria are as follows:

- ➢ Studies that focus on palliative care quality measures on cancer patients in LMICs
- ➢ Study articles published any period before 31 July 2022
- ➢ Qualitative, quantitative and mixed methods studies
- ➢ Studies written in English

### Exclusion criteria

The exclusion criteria are as follows:

- ➢ Studies of palliative care quality measures on any patients other than cancer patients
- ➢ Review studies
- ➢ Studies written in any other language but English

**Fig. 1:**
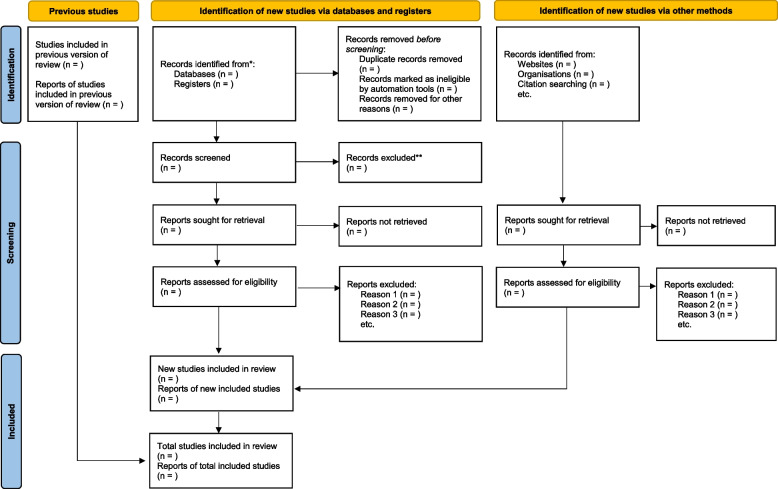
PRISMA flow diagram of the study selection process. Single asterisk (*) symbol indicates the following: consider, if feasible to do so, reporting the number of records identified from each database or register searched (rather than the total number across all databases/registers). Double asterisk (**) symbol indicates the following: if automation tools were used, indicate how many records were excluded by a human and how many were excluded by automation tools

All eligible articles will be exported to the Rayyan software [23], and duplicates will be removed. The articles will be screened in three phases: phase 1—comprehensive article search using title (title screening), phase 2—abstract screening and phase 3—full-article screening. The PI and co-authors will screen titles and abstracts in parallel. After screening, the reviewers will discuss any discrepancies in selected articles until a consensus has been reached. An independent reviewer will be reached in the event were the co-authors do not reach a consensus on selected articles. When a consensus has been met, the PI and co-authors will conduct full article screening of selected articles. Both abstract and full article screening will be guided by the inclusion/exclusion criteria. We will report the screening results following the PRISMA guidelines (Fig. 1).

### Charting the data

A charting form to capture information from each relevant study was developed. The PI and the co-author will pilot and modify the data charting form before commencing the scoping review. The form that will be used for data charting is presented in Table 3.

**Table 3:** Data charting form

**Author and date**
Study title
Author and year of publication
Journal full reference
Aims or study objective(s)
study setting and geography
Study design
Data collection methods
Data analysis
Relevant findings
Outcomes
Conclusion
Comments

### Collating, summarising and reporting the results

A narrative report will be compiled to summarise analysed extracted data according to themes. The themes will be structured around the following outcomes: region of study, study period, categories of quality measures, structure and processes of care. These results will be described in relation to the research question and in the context of the overall study purpose. All themes including emerging themes will be corresponded according to respective authors. The researchers will use google forms and NVivo version 12 for thematic analysis. The following is the process to be followed:

- ➢ Coding
- ➢ Categorise codes into major themes
- ➢ Build theme-related themes using the cut and paste technique
- ➢ Display of data
- ➢ Identification of patterns and sub-themes in the data
- ➢ Summarising

### Quality appraisal

To assess the quality of the included studies, we will use the mixed methods appraisal tool (MMAT), version 2018 [24]. The quality appraisal procedure will be carried out by two impartial reviewers. The quality of the evidence will be graded using the following percentage scores: (1) 50% will represent low quality evidence, (2) 51–75% will represent average quality evidence, and (3) 76–100% will represent good quality evidence. We will be able to evaluate various study methodologies, such as qualitative, quantitative, or mixed-methods studies, using this quality appraisal method.

## Conclusion

The purpose is to establish the extent of existing research on the palliative care quality measures in LMICs. Although palliative care is still a new phenomenon, understanding of the palliative care quality measures applicable for cancer patients will assist to improve care across all components of health systems.

## Data Availability

All data generated or analysed during this study will be included in the published scoping review article.

## Abbreviations

LMICs: Low- and middle-income countries
HIC: High-income countries
WHO: World Health Organization
MMAT: Mixed methods appraisal tool
PI: Principal investigator
PRISMA: Preferred Reporting Items for Systematic Reviews and Meta-analyses
MeSH: Medical Subject Headings

## References

[1] Anand P, Kunnumakara AB, Sundaram C, Harikumar KB, Tharakan ST, Lai OS (2008). Cancer is a preventable disease that requires major lifestyle changes. Pharm Res.

[2] World Health Organization. Estimated number of new cases from 2020 to 2040, both sexes, age [0–85+]. International Agency for Research of Cancer; 2020. Available from: https://gco.iarc.fr/tomorrow/en/dataviz/isotype?types=0&single_unit=500000. Cited 2021 Jan 10.

[3] National Cancer Institute. Cancer statistics. National Cancer Institute; 2020. Available from: https://www.cancer.gov/about-cancer/understanding/statistics#:~:text=The%20rate%20of%20new%20cases,on%202013%E2%80%932017%20deaths).

[4] Worldwide Palliative Care Alliance. Global atlas of palliative care at the end of life. In: Connor SR, Bermedo MCS, editors. London: World Health Organization; 2014. p. 1–111.

[5] World Health Organization. Sixty-seventh world health assembly strengthening of palliative care as a component of comprehensive care throughout the life course. Geneva: World Health Organization; 2014.

[6] World Health Organization. Fifty-eighth world health assembly. 2005.

[7] World Health Organization. Palliative care. Newsroom Fact Sheet; 2020. Available from: www.who.int/news-room/fact-sheets/detail/palliative-care. Cited 2020 Oct 28.

[8] Downing J, Grant L, Leng M, Namukwaya E (2015). Understanding models of palliative care delivery in sub-Saharan Africa: learning from programs in Kenya and Malawi. J Pain Symptom Manage.

[9] Robertson J, Barr R, Shulman LN, Forte GB, Magrini N (2016). Essential medicines for cancer: WHO recommendations and national priorities. Bull World Health Organ.

[10] Pace L, Schleimer L, Shyirambere C, Illawi A, Dusengimana J, World Health Organization (WHO) (2020). Identifying breast cancer care quality measures for a cancer facility in rural Sub-Saharan Africa: results of a systematic literature review and modified ed delphi process. Am Soc Clin Oncol.

[11] Tieman J, Sladek R, Currow D (2008). Changes in the quantity and level of evidence of palliative and hospice care literature: the last century. J Clin Oncol.

[12] Abu-Odah H, Molassiotis A, YatWa Liu J (2022). Global palliative care research (2002–2020)- bibliometric review and mapping analysis. BMJ Support Palliat Care.

[13] Gwyther L, Krakauer E. WPCA policy statement on defining palliative care. London: The Worldwide Palliative Care Alliance (WPCA); 2013. p. 1–6.

[14] Reville B, Foxwell AM (2014). The global state of palliative care — progress and challenges in cancer care. Ann Palliat Med.

[15] Mwangi-Powell F, Dix O (2011). Palliative care in Africa: an overview.

[16] García-Pérez L, Linertová R, Martín-Olivera R, Serrano-Aguilar P, Benítez-Rosario MA (2009). A systematic review of specialised palliative care for terminal patients: which model is better?. Palliat Med.

[17] Kamal AH, Gradison M, Maguire JM, Taylor D, Abernethy AP (2014). Quality measures for palliative care in patients with cancer: a systematic review. Proc Am Soc Clin Oncol.

[18] Kamal AH (2013). Time to define high-quality palliative care in oncology. J Clin Oncol.

[19] Kamal AH, Bausewein C, Casarett DJ, Currow DC, Dudgeon DJ, Higginson IJ (2020). Standards, guidelines, and quality measures for successful specialty palliative care integration into oncology: current approaches and future directions. J Clin Oncol.

[20] The World Bank. The World Bank in middle income countries. 2022. Available from: www.worldbank.org/en/country/mic/overview#:~:text+They%20are%20defined%20as%20lower,62%25%20of%20the%20world’s%20poor. Cited 2023 Mar 16.

[21] Arksey H, O’Malley L (2005). Scoping studies: towards a methodological framework. Int J Soc Res Methodol Theory Pract.

[22] Levac D, Colquhoun H, O’Brien KK (2010). Scoping studies: advancing the methodology. Implement Sci.

[23] Ouzzani M, Hammady H, Fedorowicz Z, Elmagarmid A. Rayyan-a web and mobile app for systematic reviews. In: Systematic reviews. Vol. 5. BioMed Central Ltd.; 2016. Available from: rayyan.ai/cite/. Cited 2023 Mar 22. 10.1186/s13643-016-0384-4 PMC5139140 27919275

[24] Hong Q, Pluye P, Fàbregues S, Bartlett G, Boardman F, Cargo M, et al. Mixed Methods Appraisal Tool (MMAT): user guide. McGill. Canadian Intellectual Property Office, Industry Canada. 2018. p. 1–11.

